# Inhibition of Gap Junction Communication at Ectopic Eph/ephrin Boundaries Underlies Craniofrontonasal Syndrome

**DOI:** 10.1371/journal.pbio.0040315

**Published:** 2006-09-12

**Authors:** Alice Davy, Jeffrey O Bush, Philippe Soriano

**Affiliations:** Program in Developmental Biology, Division of Basic Sciences, Fred Hutchinson Cancer Research Center, Seattle, Washington, United States of America; California Institute of Technology, United States of America

## Abstract

Mutations in X-linked *ephrin-B1* in humans cause craniofrontonasal syndrome (CFNS), a disease that affects female patients more severely than males. Sorting of ephrin-B1–positive and –negative cells following X-inactivation has been observed in *ephrin-B1^+/−^* mice; however, the mechanisms by which mosaic *ephrin-B1* expression leads to cell sorting and phenotypic defects remain unknown. Here we show that *ephrin-B1^+/−^* mice exhibit calvarial defects, a phenotype autonomous to neural crest cells that correlates with cell sorting. We have traced the causes of calvarial defects to impaired differentiation of osteogenic precursors. We show that gap junction communication (GJC) is inhibited at ectopic ephrin boundaries and that ephrin-B1 interacts with connexin43 and regulates its distribution. Moreover, we provide genetic evidence that GJC is implicated in the calvarial defects observed in *ephrin-B1^+/−^* embryos. Our results uncover a novel role for Eph/ephrins in regulating GJC in vivo and suggest that the pleiotropic defects seen in CFNS patients are due to improper regulation of GJC in affected tissues.

## Abstract 2

Mutations in*ephrin-B1* associated with X-linked CFNS result in aberrant formation of facial bone structures.*Ephrin-B1* interacts with gap junctions to influence the cell sorting events that underlie the CFNS deformations.

## Introduction

Physical segregation, or sorting, of different cell populations during development is essential for the proper spatial organization of the animal body. Eph receptor tyrosine kinases and ephrins regulate many developmental processes [1–3], and play an important role in tissue patterning by restricting cell intermingling and establishing developmental boundaries [4–6].

A dramatic example of the role of Eph and ephrins in cell sorting, as well as the importance of proper cell sorting during development, was recently provided by the analysis of the phenotypes exhibited by *ephrin-B1* heterozygous female mice [7,8] and by the identification of mutations in the *ephrin-B1* gene in human craniofrontonasal syndrome (CFNS) patients [9,10]. As a result of random X-inactivation, X-linked *ephrin-B1* expression is mosaic in *ephrin-B1^+/−^* mice and ephrin-B1–positive and ephrin-B1–negative cells segregate from one another. This correlates with a polydactyly phenotype that is never observed in *ephrin-B1* null animals [7,8]. Similarly, CFNS is an X-linked developmental disorder characterized by a number of craniofacial defects including abnormal development of the cranial and nasal bones, and craniosynostosis (premature fusion of the coronal sutures), as well as extracranial anomalies (including polydactyly and syndactyly), affecting mainly female patients [11].

Despite years of intensive studies, the molecular mechanisms by which Eph receptors and ephrins influence cell sorting are still poorly understood. Eph receptors and ephrins function as an unusual receptor/ligand pair in which both receptor and ligand are capable of activating a signaling cascade. One prominent outcome of Eph/ephrin interactions is the regulation of cell–substrate adhesion and reorganization of the actin cytoskeleton. It has also been reported that Eph receptors and ephrins regulate gap junction communication (GJC) [6]. Indeed, studies in zebrafish have shown that expression of Eph receptors and ephrins in animal cap cells was sufficient to block GJC at the boundary between both cell populations. Gap junctions are intercellular membrane channels that mediate cell coupling by allowing the passage of small molecules directly from cell to cell. During vertebrate development, regions of GJC coincide with developmental compartments [12,13]. For instance, GJC is reduced at inter-rhombomeric boundaries, as compared to GJC in the rhombomeres themselves. GJC is involved in various developmental processes and mutations in connexins, the structural proteins forming gap junctional pores, have been linked to a number of human diseases [14]. Notably, GJC plays an important role in skeletal development [15] and mutations in connexin43 (Cx43) lead to cranial and skeletal defects both in humans and mice [16,17].

In this report, we have investigated the underlying cause of the phenotypes observed in *ephrin-B1* heterozygous mice. We show that cell sorting in *ephrin-B1^+/−^* females induces calvarial defects due to the impaired differentiation of neural crest cells (NCCs). We provide evidence that GJC is inhibited at ectopic ephrin boundaries and that ephrin-B1 physically interacts with Cx43 and influences its distribution. In addition, we report that overexpression of Cx43 partially rescues the calvarial defects observed in *ephrin-B1* heterozygotes. Finally, we show that regulation of GJC correlates with cell sorting in response to Eph/ephrin interaction. From these results we conclude that mosaic loss of *ephrin-B1* exerts a dominant effect during development involving perturbation of GJC at ectopic Eph/ephrin boundaries and leading to defective tissue differentiation. These observations extend our understanding of the mechanisms underlying CFNS in humans and of the role of Eph receptors and ephrins in vivo.

## Results

### Craniofacial Phenotypes in *ephrin-B1* Heterozygous Females

**Figure 1:**
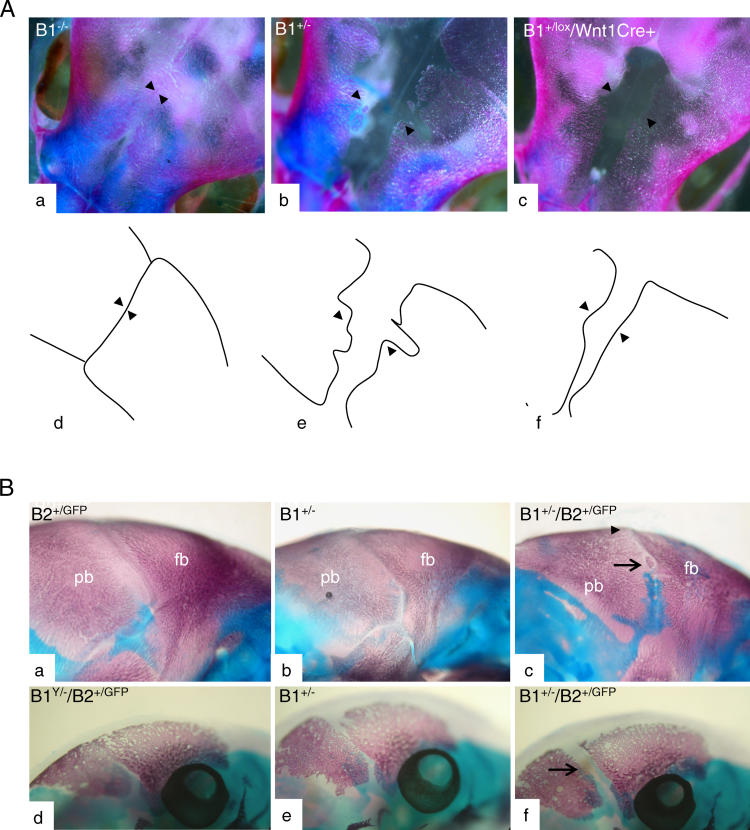
*ephrin-B1^+/−^* Embryos Exhibit Defects in NCC Derivatives (A) Skeleton preparations of whole heads from E18.5 mutant embryos (a–c). Bones are stained with Alizarin Red while cartilage is stained with Alcian Blue. An opening (foramen) between frontal bones is observed in *ephrin-B1^+/−^* heterozygous females (b) but not in *ephrin-B1^−/−^* homozygous females (a). Mutant embryos in which ephrin-B1 is specifically deleted in NCC also exhibit defects of the frontal bones (c). Arrowheads show the bone front of the frontal bones. Schematic drawings of the frontal bones (d–f). (B) Skeleton preparations of whole heads from E18.5 embryos (a–c) or E15.5 embryos (d–f) show that *ephrin-B1^+/−^/ephrin-B2^+/GFP^* embryos present an additional coronal suture defect (c) and (f) as compared to *ephrin-B1^+/−^* single heterozygous females (b) and (e). At E18.5, bone fronts never overlap at coronal sutures of *ephrin-B1^+/−^/ephrin-B2^+/GFP^* embryos, and ectopic bone forms in the suture mesenchyme (arrow in [c]). At E15.5, parietal and frontal bone fronts forming the coronal suture (arrow) of *ephrin-B1^+/−^/ephrin-B2^+/GFP^* embryos (f) are further apart than in control littermates (d) and (e). Coronal suture and frontal bones are normal in single *ephrin-B2^+/GFP^* heterozygous mutants (a) and in *ephrin-B1^Y/−^/ephrin-B2^+/GFP^* males (d). fb, frontal bone; pb, parietal bone.

We and others have shown previously that *ephrin-B1^+/−^* females exhibit a polydactyly phenotype that is never seen in *ephrin-B1^Y/−^* males or *ephrin-B1^−/−^* females [7,8]. Because CFNS human female patients exhibit numerous craniofacial defects, we undertook a closer analysis of *ephrin-B1* heterozygous female mice that revealed several defects in NCC-derived tissues that were not seen in hemizygous males or homozygous females. At P1, *ephrin-B1^+/−^* heterozygous females (*n* = 17) presented an opening (foramen) between the frontal bones and jagged bone fronts (Figure 1Ab), indicative of abnormal development of the frontal bones. (The parietal bone was affected at lower penetrance). *Ephrin-B1^−/−^* homozygous null females (*n* = 3) and *ephrin-B1^Y/−^* hemizygous males (*n* = 13) exhibited a normal development of these bones (Figure 1Aa and unpublished data). The frontal bone phenotype was recapitulated in heterozygous females carrying a deletion of *ephrin-B1* specifically in NCCs (*n* = 3) (Figure 1Ac), consistent with the NCC origin of frontal bones [18]. *Ephrin-B1^+/−^* females also exhibited abnormal alignment of the vibrissae buds which was recapitulated by conditional deletion of *ephrin-B1* in NCCs *(ephrin-B1^+/lox^;Wnt1Cre+),* indicating that the defect is autonomous to this lineage (Figure S1).

**Figure 2:**
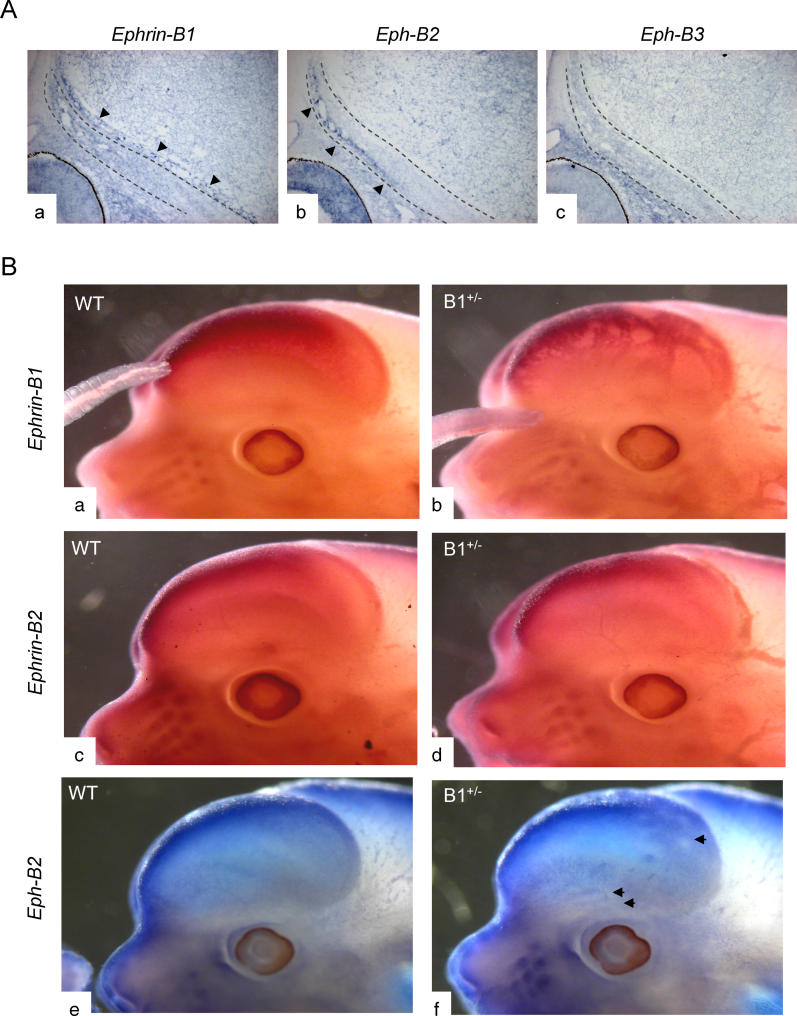
Expression of *ephrin-B1* and *EphB2* Is Abnormal in *ephrin-B1^+/−^* Embryos (A) In situ hybridization on frontal sections of E14.5 wild-type embryo using probes for *ephrin-B1* (a), *EphB2* (b), or *EphB3* (c). Ephrin-B1 is expressed throughout the developing frontal bone (marked by dotted lines) as well as in the meningeal layer (arrowheads). Expression of EphB2 is restricted ventrally (arrowheads) whereas EphB3 is expressed throughout the developing frontal bone. (B) In situ hybridization of wild-type (a), (c), and (e) or *ephrin-B1^+/−^* embryos (b), (d), and (f) using a probe for *ephrin-B1* (a) and (b), *ephrin-B2* (c) and (d) and *EphB2* (e) and (f). Both ephrin-B1 and EphB2 show sorting in the telencephalon and the craniofacial mesenchyme of *ephrin-B1^+/−^* embryos (arrowheads) while expression of ephrin-B2 is unaffected.

It has been proposed by others that the mild manifestations of CFNS in male carriers might be due to compensatory mechanisms by other ephrins [10]. To test this hypothesis, we asked whether further alteration in the dosage of ephrin signaling would lead to calvarial phenotypes in *ephrin-B1* null mutants. We chose to focus on *ephrin-B2* since loss of this gene has been shown previously to affect migration of a sub-population of cranial NCCs [19] and *ephrin-B2* is expressed in the craniofacial area (Figure 2) We generated a mouse line harboring a null mutation in the *ephrin-B2* gene by placing a cDNA coding for a fusion protein between histone 2B (H2B) and the green fluorescent protein (GFP) under the control of the *ephrin-B2* promoter (A. Davy, P. Soriano, unpublished data). *ephrin-B2* and *ephrin-B1* heterozygous animals were mated to generate *ephrin-B1/ephrin-B2* double heterozygous animals. Skeleton preparations showed that removal of one copy of *ephrin-B2* in an *ephrin-B1^+/−^* background worsened the calvarial phenotype (Figure 1B). Indeed, in addition to a larger gap between the frontal bones (Figure S2A), *ephrin-B1^+/−^/ephrin-B2^+/GFP^* embryos (*n* = 5) exhibited a coronal suture defect that was not observed in *ephrin-B1* single mutants, males or females. Bone fronts at coronal sutures in *ephrin-B1^+/−^/ephrin-B2^+/GFP^* embryos did not overlap, and ectopic bone was frequently observed in the suture mesenchyme (Figure 1Bc). Examination of skeleton preparations from E15.5 embryos indicated that although bone formation in *ephrin-B1^+/−^/ephrin-B2^+/GFP^* embryos proceeded comparably to *ephrin-B1^+/−^* embryos, bone fronts seemed unable to extend toward each other at the coronal suture (Figure 1Bf). Importantly, *ephrin-B1^Y/−^/ephrin-B2^+/GFP^* double hemi/heterozygous males (*n* = 3) exhibited normal calvarial development (Figure 1Bd and unpublished data). Altogether, these results show that mosaic loss of *ephrin-B1* exerts a dominant effect on the development of calvarial bones. Removal of one copy of *ephrin-B2* in an *ephrin-B1^+/−^* background was sufficient to uncover additional defects at the coronal suture; however, it did not lead to calvarial phenotypes in *ephrin-B1* null embryos, suggesting that the lack of calvarial phenotype in these embryos is not due to compensation by *ephrin-B2*.

### Defects in NCC-Derived Structures Correlate with Cell-Sorting in *ephrin-B1^+/−^* Embryos

To better understand the basis for the calvarial phenotypes observed in *ephrin-B1* heterozygotes, we analyzed the expression pattern of *ephrin-B1* and *ephrin-B2,* and their cognate receptors *EphB2* and *EphB3* at various stages of development. These receptors have been implicated both in the polydactyly phenotype and in the formation of the palate [20], a NCC-derived structure that also requires *ephrin-B1.* At E14.5, expression of *ephrin-B1, EphB2,* and *EphB3* can be detected in the developing frontal bones (Figure 2A). *Ephrin-B1* and *EphB3* are expressed throughout the bone whereas *EphB2* expression appears to be restricted ventrally. Interestingly, *ephrin-B1* is also strongly expressed in the meningeal layer which derives from neural crest cells [18]. At E12.5, a stage that corresponds to the early stages of calvarial bone differentiation, *ephrin-B1* is expressed throughout the head mesenchyme as well as in the vibrissae buds in wild-type embryos (Figure 2Ba and Figure S1). In *ephrin-B1^+/−^* embryos, expression of *ephrin-B1* is patchy throughout the craniofacial mesenchyme and in the telencephalon, and highlights the defective formation of vibrissae buds (Figure 2Bb). Both *ephrin-B2* and *EphB2* exhibit expression patterns that are similar to *ephrin-B1* (Figure 2Bc and 2Be). Expression of *ephrin-B2,* however, is unchanged in *ephrin-B1^+/−^* (Figure 2Bd) whereas *EphB2* expression appears patchy (Figure 2Bf). It has been reported previously that patchy expression of *ephrin-B1* in *ephrin-B1^+/−^* limb buds reflects sorting between ephrin-B1–positive and –negative cell populations that are generated in the *ephrin-B1* heterozygous females via random X-inactivation and that this abnormal expression of ephrin-B1 in the limb bud correlates with a polydactyly phenotype that is observed in *ephrin-B1^+/−^* females [7,8]. Our data demonstrate that the calvarial phenotypes observed in *ephrin-B1* heterozygous females correlate with an abnormal expression of *ephrin-B1* and *EphB2* in the presumptive frontal bone, likely due to cell sorting between ephrin-B1–positive and ephrin-B1–negative cells in the craniofacial mesenchyme.

### The Frontal Bone Defect Is Caused by Abnormal Osteogenic Differentiation

**Figure 3:**
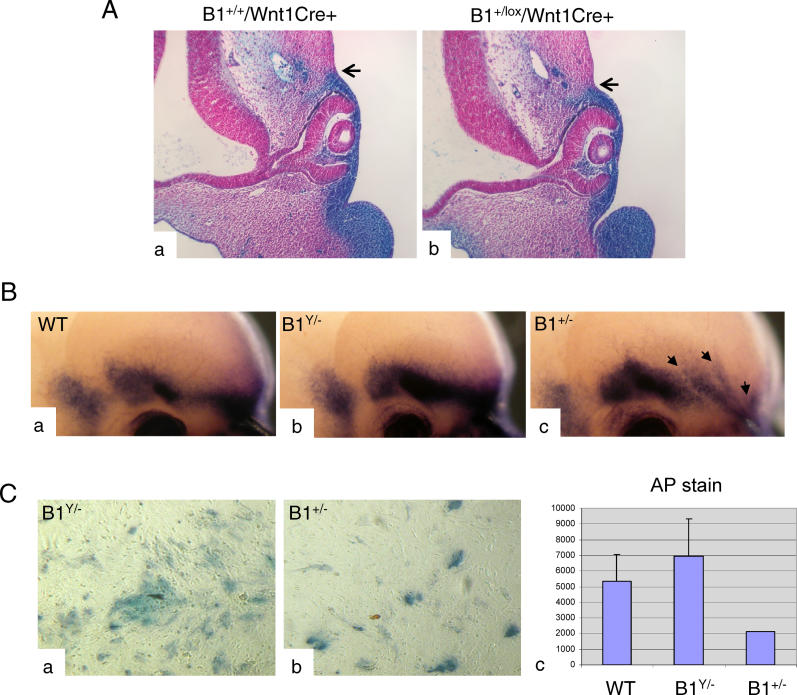
Calvarial Foramen Correlates with Impaired Osteogenic Differentiation (A) E11.5 embryos carrying the *R26R* and *Wnt1-Cre* alleles were processed for X-gal staining to label NCCs. No difference in the osteogenic precursor population (arrow) was detected in *ephrin-B1^+/−^* females (b) as compared to wild type (a). (B) E12.5 wild-type (a), *ephrin-B1^Y/−^* (b), and *ephrin-B1^+/−^* (c) embryos were stained for AP activity. The onset of osteogenic differentiation does not seem affected in the mutant embryos, but AP staining pattern is irregular in the heterozygote mutant as compared to wild-type and homozygote mutant, with areas devoid of staining (arrows). (C) Primary mesenchymal cells were isolated from presumptive calvaria of E14.5 *ephrin-B1^Y/−^* hemizygous male (a) or *ephrin-B1^+/−^* heterozygous female (b) and plated at high density. AP activity was evaluated after 3 d in culture. Digital quantification of AP activity (c).

To uncover the nature of the dominant effect of mosaic loss of *ephrin-B1,* we reasoned that understanding why sorting-out between ephrin-B1–positive and ephrin-B1–negative cells has such consequences for the development of this tissue would shed light on the dominant function of ephrin-B1. NCCs are the source of the frontal bone osteoprogenitor population, and both *ephrin-B1* and *ephrin-B2* control migration of these cells, raising the possibility that improper migration of NCC progenitors could be responsible for the frontal bone phenotype. Using a combination of *Wnt1Cre/R26R* alleles to specifically label NCCs, we found no difference in the size of the progenitor pool between *ephrin-B1* heterozygous females and wild-type animals (Figure 3A), indicating that defective migration is not the likely cause for the frontal bone phenotype. In addition, no proliferation or cell survival defects were detected on sections of mutant frontal bones (unpublished data).

To test whether the defects in calvarial bone development in *ephrin-B1^+/−^* embryos correlated with perturbation of osteoblastic differentiation, we used alkaline phosphatase (AP) activity as a marker of early osteoblastic differentiation. At E16.5, AP staining of frontal bones showed delayed differentiation in *ephrin-B1^+/−^/ephrin-B2^+/GFP^* and *ephrin-B1^+/−^* (Figure S2B and unpublished data). At E12.5, similar levels of AP activity could be detected in wild-type, *ephrin-B1^Y/−^,* and *ephrin-B1^+/−^* embryos (Figure 3B), indicating that defective bone growth was not due to delayed onset of differentiation in heterozygous mutants. However, unlike wild-type and *ephrin-B1^Y/−^* embryos which showed continuous AP activity, AP staining of the presumptive frontal bone appeared irregular in *ephrin-B1* heterozygous embryos (Figure 3Bc). These observations indicate that the calvarial defects observed in *ephrin-B1^+/−^* mutants are not due to abnormal migration or survival of NCCs, but instead might be due to a defective differentiation of the presumptive osteogenic mesenchyme.

To confirm the differentiation defects observed in *ephrin-B1* heterozygotes, we isolated presumptive osteogenic mesenchymal cells from E14.5 wild-type and *ephrin-B1^+/−^* embryos, and evaluated their ability to differentiate in vitro. Using AP activity as a marker for osteogenic differentiation, we observed that cells isolated from *ephrin-B1^+/−^* embryos were consistently less prone to differentiate in vitro (Figure 3C), indicating that these defects are autonomous to the osteoprogenitor cells. In these primary cultures, expression of ephrin-B1 was detected in a punctate pattern in both AP-positive as well as AP-negative cells (Figure S3A), indicating that expression of ephrin-B1 and AP do not strictly correlate, and suggesting that ephrin-B1 does not regulate AP activity directly.

### Defective Osteogenic Differentiation in *ephrin-B1^+/−^* Embryos Correlates with Abnormal Cx43 Distribution

**Figure 4:**
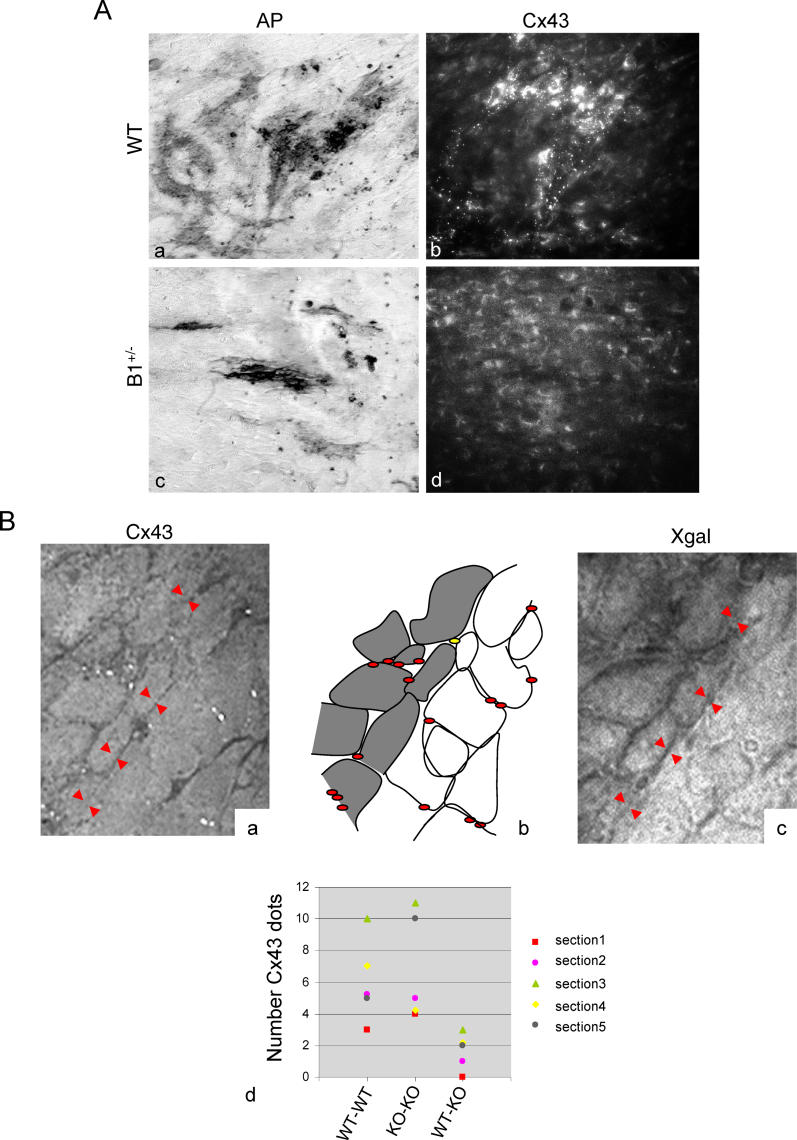
Decreased Osteogenic Differentiation Correlates with Abnormal Distribution of Cx43 (A) Primary mesenchymal cells isolated from presumptive calvaria of wild-type (a) and (b) or *ephrin-B1^+/−^* embryos (c) and (d) were stained for AP activity (a) and (c) and subsequently processed for Cx43 immunofluorescence (b) and (d). Junctional Cx43 evidenced by bright dots is readily detected in cultures from wild-type embryos (WT), but not in cultures from *ephrin-B1^+/−^* embryos. (B) Detection of Cx43 by immunofluorescence in limb bud sections of an X-gal–stained chimeric embryo obtained by injecting ephrin-B1 null cells into a ROSA26-βgal blastocyst. Gap junctional Cx43 appears as bright dots (a). The boundary between wild-type cells (dark cells in [c]) and *ephrin-B1* null cells (white cells in [c]) is indicated by red arrows. A schematic representation of the results is shown (b). Grey cells are wild type and white cells are ephrin-B1 null. Cx43 dots are in red, whereas yellow in (b) marks the only Cx43 dot that might be between a wild-type and a null cell. Quantification of Cx43 dots in multiple sections show a reduction in the number of Cx43 dots between wild-type and null cells (WT-KO) (d).

Using this in vitro system, we tested a number of markers that have been shown to regulate osteogenic differentiation, including N-cadherin and Cx43 expression, as well as activation of MAPK. Among these, only Cx43 distribution showed prevalent changes between cultures from wild-type and *ephrin-B1* heterozygous embryos (Figure 4A and unpublished data). Cx43 is a structural protein that forms gap junctional pores (connexons). Whereas cytoplasmic Cx43 shows a diffuse staining by immunofluorescence, cell surface connexons appear as bright dots because they aggregate to form functional gap junctional plaques at cell–cell interfaces. In cultures of mesenchymal cells isolated from wild-type embryos, the differentiating cells expressed a high level of Cx43, and gap junctional plaques were readily visible between these cells (Figure 4Aa and 4Ab). On the contrary, in cultures of mesenchymal cells isolated from *ephrin-B1* heterozygous embryos, the distribution of Cx43 was altered and gap junctional plaques were not detected (Figure 4Ac and 4Ad). Western blot analysis indicated that the overall level of Cx43 was unchanged in primary cultures isolated from *ephrin-B1* heterozygous embryos, indicating that the distribution but not the expression of Cx43 was altered in primary cultures from *ephrin-B1^+/−^* embryos (Figure S3B).

We next tested whether distribution of Cx43 was also altered in *ephrin-B1* mosaic embryos. For this purpose, we co-stained paraffin sections from control and heterozygous mutant E12.5 embryos with AP and Cx43. However, even though AP staining of the frontal bone was very irregular in the *ephrin-B1^+/−^* embryo, compared to the control frontal bone (Figure S3Ca and S3Cc), we were unable to detect significant differences in Cx43 staining on these sections (Figure S3Cb and S3Cd). Since *ephrin-B1* null embryos do not show calvarial phenotype or abnormal osteogenic differentiation, we reasoned that ephrin-B1 itself might not be required for proper localization of Cx43, but rather that abnormal distribution of Cx43 might be seen only at the boundary between ephrin-B1–positive and –negative cells in the mosaic embryos. To be able to detect boundaries between ephrin-B1–positive and ephrin-B1–negative cells, we generated chimeric embryos by injecting *ephrin-B1* null embryonic stem (ES) cells in wild-type ROSA26 blastocysts which express β-galactosidase constitutively. Paraffin sections of X-gal–stained E11.5 chimeric embryos were processed for immunofluorescence using the Cx43 antibody. Gap junctional Cx43 was readily detected between wild-type cells and between *ephrin-B1* null cells (Figure 4B). However, gap junctional Cx43 was almost never observed between ephrin-B1–positive and –negative cells. We concluded that the number of junctional pores is diminished at ephrin-B1–positive/ephrin-B1–negative boundaries in vivo.

### Ephrin-B1 Associates with Cx43 and Regulates GJC

**Figure 5:**
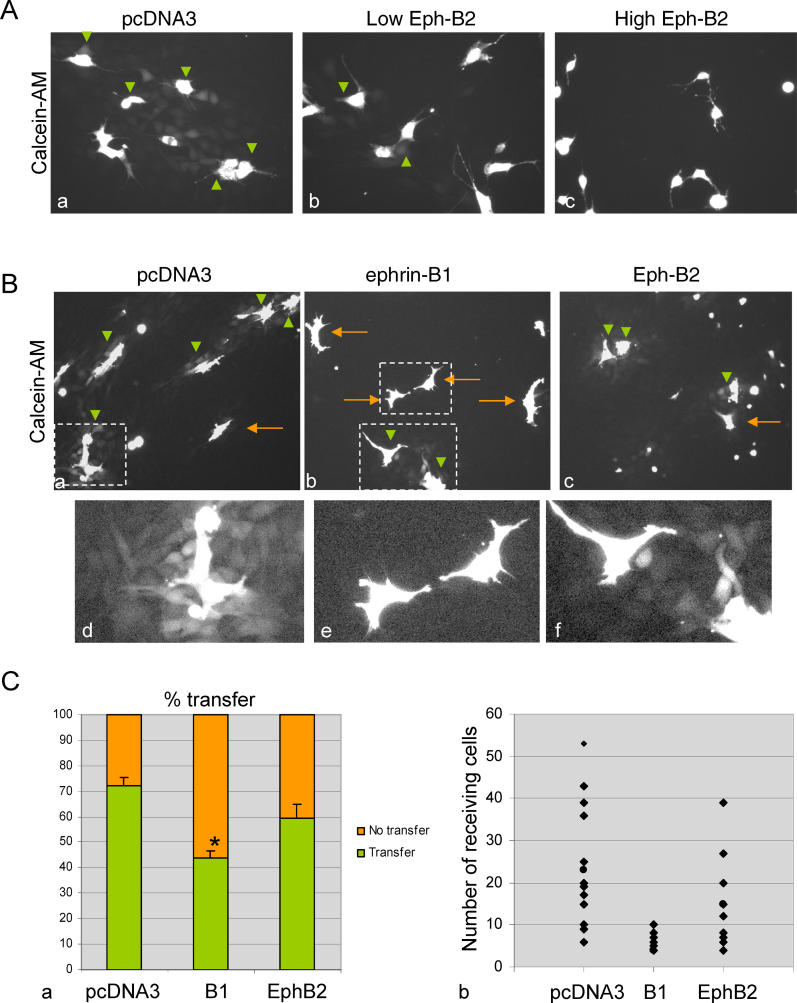
Eph/ephrin Interaction Inhibits Gap Junction Communication (A) NIH 3T3 cells stably expressing ephrin-B1 were loaded with calcein-AM and dropped onto monolayers of NIH 3T3 cells either not expressing (a) or expressing variable levels (b) and (c) of Eph-B2 receptor. Transfer of dye to neighboring cells (arrowheads) was evaluated by fluorescence after 3 h. No dye transfer (arrows) was observed when high levels of Eph-B2 were expressed. (B) Primary NCCs were loaded with Calcein-AM and dropped onto NIH 3T3 cells that were transfected either with a control plasmid (pcDNA3 [a]), or an expression construct for ephrin-B1 (b) or Eph-B2 (c). In the control situation, the majority of cells transferred the dye (arrowheads). The boxed area is shown at higher magnification in (d). Transfer of dye is visualized by faint staining of cells surrounding the very bright donor cell. Following Eph/ephrin interaction, many of the donor cells did not transfer the dye (arrows). Boxed areas in (b) are shown at higher magnification in (e) and (f). Cells transferring the dye (f) and cells not transferring the dye (e) are able to spread. (C) The percentage of cells that had spread and showed transfer of dye was evaluated after 3 hours (a). In addition, the number of receiving cells for each donor cell was counted (b). *p < 0.05 compared to control.

Compagni et al. have shown that the levels of EphB2 receptor are up-regulated in ephrin-B1–negative domains in *ephrin-B1^+/−^* embryos, thus creating ectopic Eph/ephrin boundaries [7]. We therefore reasoned that decreased junctional Cx43 at ephrin-B1–positive/ephrin-B1–negative boundaries in vivo could in fact indicate an inhibition of GJC by Eph/ephrin signaling. To test whether Eph/ephrin signaling could regulate GJC, we used calcein-AM as a marker of GJC in vitro. We found that interaction between ephrin-B1 and Eph-B2 resulted in inhibition of GJC in vitro (Figure 5). NIH 3T3 cells expressing ephrin-B1 showed reduced transfer of calcein-AM when plated on cells expressing low levels of Eph-B2 (Figure 5Ab), as compared to control cells (Figure 5Aa). More dramatically, plating of ephrin-B1–expressing cells over cells expressing high levels of Eph-B2 resulted in a complete inhibition of GJC (Figure 5Ac). Similar results were obtained using primary NCCs that express both ephrin-B1 and Eph-B2 at low levels. Although primary NCCs were able to establish strong GJC with control fibroblasts (Figure 5Ba and 5Bd), plating onto fibroblasts expressing ephrin-B1 markedly decreased dye transfer, even though the majority of the cells were able to spread normally (Figure 5Bb and 5Be). The fact that some cells spread normally, but were unable to transfer the dye (Figure 5Bf), indicates that inhibition of GJC is not due to cell repulsion. Plating of primary NCCs onto fibroblasts expressing Eph-B2 resulted in a moderate reduction in GJC (Figure 5Bc). We quantified GJC by two means: first, we evaluated the number of donor cells that had spread and were able to transfer the dye (Figure 5Ca). Second, we counted the number of receiving cells for each donor cell that had spread and transferred the dye (Figure 5Cb). Both measurements indicate that GJC is diminished when primary NCCs are plated on ephrin-B1– (and to a lower extent EphB2–) expressing cells. These results demonstrate that interaction between EphB2 and ephrin-B1 impairs establishment of GJC.

**Figure 6:**
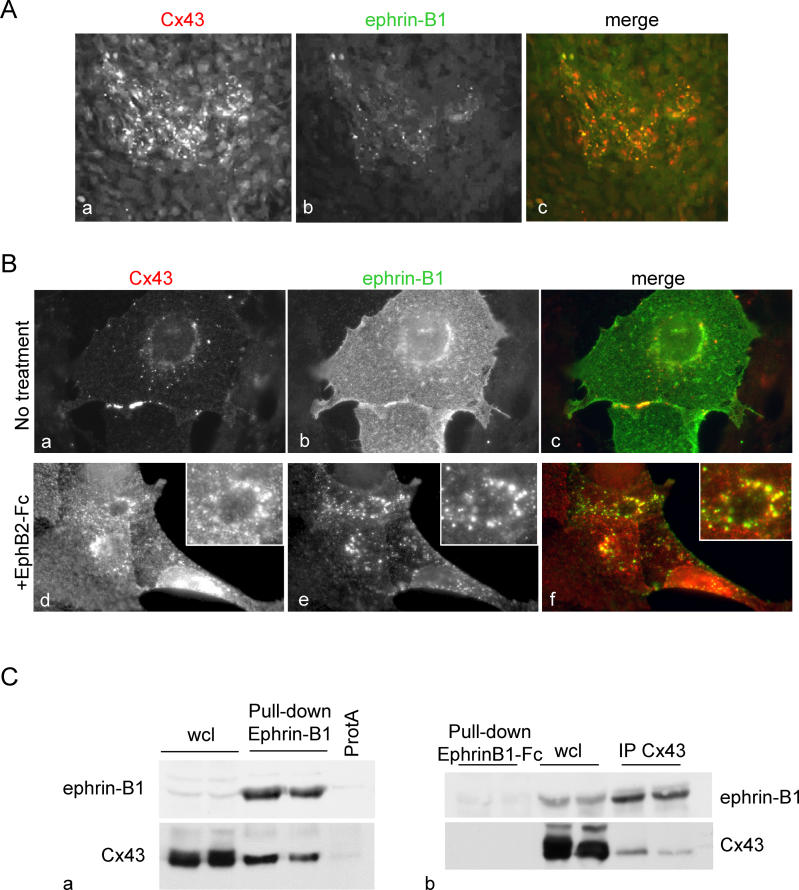
Ephrin-B1 and Cx43 Co-localize and Interact with Each Other (A) Primary mesenchymal were co-stained for Cx43 ([a], red in [c]) and ephrin-B1 ([b], green in [c]). (B) NIH 3T3 cells were transiently transfected with ephrin-B1 and co-immunofluorescence studies were performed to detect Cx43 (a) and (d) and ephrin-B1 (b) and (e) either without (a–c) or with engagement of ephrin-B1 (d–f). In untreated cells, Cx43 is enriched at interfaces between ephrin expressing cells and co-localizes with ephrin-B1 (c). Engagement by EphB2-Fc results in clustering of ephrin-B1 (e) and Cx43 partially co-localizes with ephrin-B1 clusters (f). Insets present higher magnification views. (C) Ephrin-B1 was affinity precipitated from NIH 3T3 cells using EphB2-Fc. The presence of Cx43 in the affinity complex was assessed by Western-blot (a). Protein lysates from NIH 3T3 cells expressing ephrin-B1 were incubated with ephrinB1-Fc, and the presence of Cx43 in the affinity complexes was assessed by Western blot (b) Cx43 was immunoprecipitated from NIH 3T3 cells expressing ephrin-B1 and the presence of ephrin-B1 in the immunocomplexes was detected by Western blot (right panel). IP, immunoprecipitation; Wcl, whole cell lysate.

To better understand the molecular mechanisms by which Eph/ephrins might impinge on Cx43 distribution and regulate GJC, we analyzed the distribution of both ephrin-B1 and Cx43 in cell culture. Co-immunofluorescence studies showed that Cx43 and ephrin-B1 partially co-localize in primary mesenchymal cells (Figure 6A). We then analyzed the effect of Eph/ephrin engagement on Cx43 distribution in cell lines in which ephrin-B1 was transiently transfected. Expression of ephrin-B1 and Cx43 was partially overlapping in untreated NIH 3T3 cells, especially at interfaces between cells expressing ephrin-B1, which exhibited strong Cx43 staining (Figure 6Ba–c). Following engagement by EphB2-Fc (a soluble form of EphB2 receptor), ephrin-B1 was found in clusters that partially overlapped with the Cx43 punctate staining (Figure 6Bd–f). Similar results were obtained using MDCK cells. These results demonstrate that ephrin-B1 and Cx43 partially co-localize at the subcellular level both in absence and presence of Eph/ephrin interaction. To test whether ephrin-B1 and Cx43 physically interact, we performed a pull-down assay in NIH 3T3 cells expressing ephrin-B1. We used a recombinant protein consisting of the extracellular domain of Eph-B2 receptor fused to the Fc fragment of human IgG (EphB2-Fc) to pull down ephrin-B1. Cx43 was detected in the pull down, indicating that it interacts with ephrin-B1 (Figure 6Ca). In a converse experiment, ephrin-B1 was co-immunoprecipitated with an anti-Cx43 antibody (Figure 6Cb). These results indicate that ephrin-B1 and Cx43 interact with each other.

### Regulation of GJC Underlies ephrin-Induced Cell Sorting

**Figure 7:**
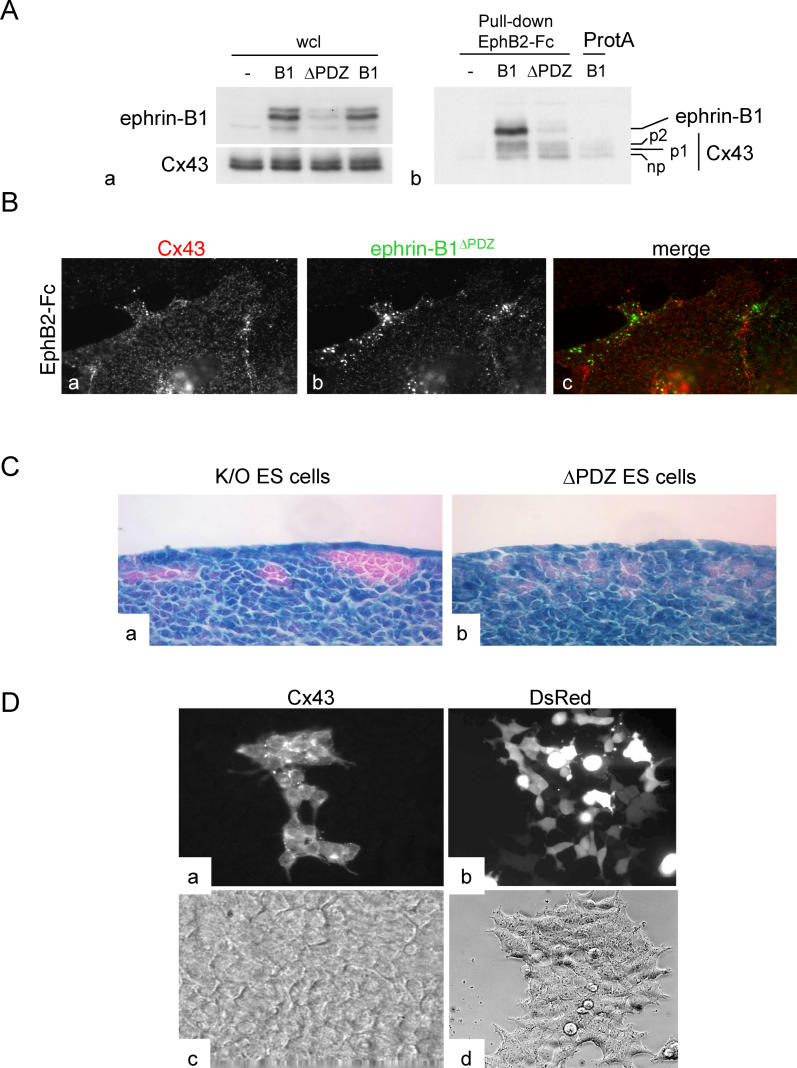
Regulation of GJC Correlates with Cell Sorting (A) NIH 3T3 cells were transiently transfected with either wild-type ephrin-B1 (B1), ephrin-B1^ΔPDZ^ (ΔPDZ), or a control plasmid (−). Protein lysates were incubated with EphB2-Fc, and the presence of Cx43 in the affinity complex was assessed by Western blot. ProtA indicates a sample that was incubated with Protein A in absence of EphB2-Fc. Whole cell lysates (a) and affinity precipitations (b) were analyzed by Western blot. np, non phosphorylated Cx43; p1, p2, two different forms of phosphorylated Cx43; wcl, whole cell lysate. (B) NIH 3T3 cells were transiently transfected with ephrin-B1^ΔPDZ^ and treated with EphB2-Fc. Subcellular localization of Cx43 (a) and ephrin-B1^ΔPDZ^ (b) was analyzed by immunofluorescence. No co-localization was observed between these two proteins (c). (C) Paraffin sections of limb buds from E11.5 chimeric embryos obtained from injection of either *ephrin-B1* null ES cells (K/O) (a) or *ephrin-B1^ΔPDZ^* ES cells (ΔPDZ) (b). Chimeric embryos were X-gal stained, processed for histology, and paraffin sections were counter-stained with Nuclear Fast Red. The wild-type cells are blue whereas the mutant cells are red. Cells expressing ephrin-B1^ΔPDZ^ do not sort from wild-type cells. (D) HEK293T cells overexpressing Cx43 were detected by immunofluorescence as a tightly packed cluster of cells (a) whereas cells expressing DsRed are scattered among non-expressing cells (b). (c) and (d) show the visible image for the same field of cells.

To identify the domain of ephrin-B1 required for the interaction with Cx43, we performed a pull down using a recombinant protein consisting of the extracellular domain of ephrin-B1 fused to the Fc fragment of human IgG (ephrinB1-Fc). Cx43 was not detected in the pull down indicating that the intracellular domain of ephrin-B1 is required for the interaction with Cx43 (Figure 6Cb). We next asked whether the PDZ-binding domain of ephrin-B1 was necessary for the interaction with Cx43, since Cx43 has been shown to interact with PDZ-containing molecules, and the data presented above suggested that Cx43 interacts with the intracellular domain of ephrin-B1. We generated a mutant form of ephrin-B1 that shows reduced binding to PDZ-containing proteins (ephrin-B1^ΔPDZ^ [8]). The ability of this mutant form of ephrin-B1 to interact with Cx43 was tested using NIH 3T3 cells that were transiently transfected with either wild-type ephrin-B1 or ephrin-B1^ΔPDZ^. Western-blot analysis of whole cell lysates indicated that both proteins were expressed, albeit at different levels, and that expression of ephrin-B1 did not influence the phosphorylation status of Cx43 (detected by differences in mobility on a SDS-PAGE), which is known to regulate GJC (Figure 7A). Cx43 could be detected in the pull downs from cells expressing either ephrin-B1 wild type or ephrin-B1^ΔPDZ^, however, the relative abundance of phosphorylated versus unphosphorylated band was changed. More phosphorylated Cx43 (slower mobility) was observed in the ephrin-B1 wild-type pull down, whereas more unphosphorylated Cx43 was detected in the ephrin-B1^ΔPDZ^ pull down (Figure 7A). These results indicate that the PDZ binding domain of ephrin-B1 is not required for its interaction with Cx43; however, ephrin-B1^ΔPDZ^ and wild-type ephrin-B1 interact preferentially with different forms of Cx43.

Because phosphorylated Cx43 is thought to represent junctional Cx43 whereas unphosphorylated Cx43 represents the inactive, cytoplasmic pool, we tested whether ephrin-B1^ΔPDZ^ co-localized with Cx43 following engagement by EphB2-Fc. Although ephrin-B1^ΔPDZ^ was present at the cell surface and localized in clusters, we did not observe co-localization of ephrin-B1^ΔPDZ^ and Cx43 following engagement by EphB2-Fc (Figure 7B). To test the effect of this mutation on calvarial development, we generated *ephrin-B1^ΔPDZ^* chimeric embryos. Examination of skeletal preparations of E18.5 *ephrin-B1^ΔPDZ^* chimeras revealed that these embryos did not exhibit defects in the calvarial bones, even in chimeras exhibiting a high degree of contribution of mutant ES cells (Figure S4), nor did they present polydactyly (unpublished data). However, subtle but consistent defects in sternum development, a phenotype that is associated with complete loss of *ephrin-B1* [7,8], were observed in almost all of the chimeras (Figure S4), indicating that the lack of calvarial and polydactyly phenotypes in the chimeric embryos is not due to an ineffective contribution of *ephrin-B1^ΔPDZ^* ES cells to bone. To test the effect of the *ephrin-B1^ΔPDZ^* mutation on the distribution of Cx43 in vivo, we generated chimeric embryos by injecting mutant ES cells carrying the *ephrin-B1^ΔPDZ^* allele (or *ephrin-B1* null ES cells as a control) in wild-type ROSA 26 blastocysts. Unexpectedly, paraffin sections of X-gal–stained E11.5 chimeric embryos revealed that unlike *ephrin-B1* null cells, cells expressing *ephrin-B1^ΔPDZ^* do not sort-out from wild-type cells (Figure 7C). These results indicate that mosaic loss of reverse signaling through the PDZ binding domain is not sufficient to drive cell sorting and does not lead to defective calvarial bone development.

The inability of ephrin-B1^ΔPDZ^ to co-localize with Cx43 upon engagement and to drive cell sorting suggested that regulation of GJC itself may play a role in the sorting-out process between ephrin-B1–positive and ephrin-B1–negative cells. To test this hypothesis, we transiently transfected HEK293T cells (that have very low levels of endogenous Cx43) with Cx43 and allowed them to sort out following trypsinization. Unlike control transfected cells, Cx43-overexpressing cells segregated from untransfected cells and were consistently found in clusters (Figure 7Da), indicating that the establishment of GJC does indeed promote cell sorting.

### Overexpression of Cx43 Partially Rescues the Calvarial Phenotype

**Figure 8:**
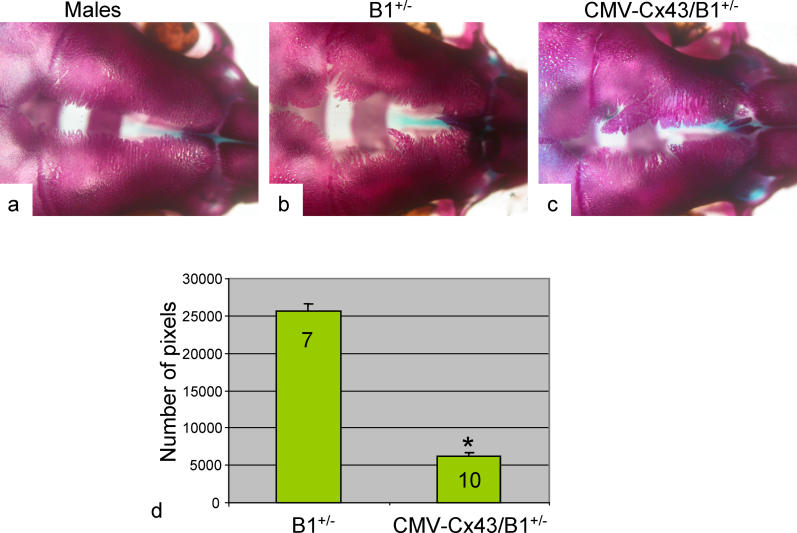
Partial Rescue of the Calvarial Defect in *ephrin-B1^+/−^* Mice Overexpressing Cx43 Skeleton preparations from an *ephrin-B1^Y/−^* male (a), an *ephrin-B1^+/−^* female (b) and an *ephrin-B1^+/−^* female carrying the *CMV-Cx43* transgene (c). Quantification (d) of the foramen area between the frontal bones of an *ephrin-B1^+/−^* female (B1^+/−^) and an *ephrin-B1^+/−^* female carrying the *CMV-Cx43* transgene *(CMV-Cx43;B1^+/−^)*. Mean values for the females with and without the transgene were normalized to the value obtained for males. The number of embryos for each genotype is indicated. Error bars represent standard error of the mean values. *p = 0.0224.

Because the lack of cell sorting in the experiments described above precluded the establishment of a functional link between the calvarial phenotype and the regulation of GJC in *ephrin-B1* heterozygote embryos, we performed a genetic rescue experiment. Mice carrying a CMV-Cx43 transgene that allows for the generalized overexpression of Cx43 [21] were bred to *ephrin-B1^−/−^* mice. Western-blot analysis showed a modest increase in the level of Cx43 in presumptive frontal bones of embryos carrying the transgene (unpublished data). Skeleton preparations of P1 offspring indicated that all of the skeletal defects previously observed in the *ephrin-B1* mutants, including the phenotype specific to *ephrin-B1* heterozygotes, were also found in this mixed genetic background (unpublished data). To assess whether overexpression of Cx43 had an effect on the calvarial phenotype observed in *ephrin-B1^+/−^* mutants, we quantified the foramen area between the frontal bones in newborn pups from all genotypes (see the Materials and Methods section). Although overexpression of Cx43 had no significant impact on the development of the frontal bones in male pups, the foramen area was decreased in the *ephrin-B1^+/−^* pups carrying the CMV-Cx43 transgene compared to *ephrin-B1^+/−^* pups without the transgene (Figure 8). Whereas the difference in foramen area between the *ephrin-B1^+/−^* pups with and without the CMV-Cx43 transgene was significant, the degree of rescue of frontal bone development in presence of the transgene was variable from sample to sample (unpublished data). Importantly, overexpression of Cx43 did not change the skeletal defects that are independent of cell sorting (i.e., sternum defects) (unpublished data). These results establish a functional link between ephrin-B1 and GJC, and suggest that improper regulation of GJC is implicated in the calvarial defects observed in *ephrin-B1* heterozygous females.

## Discussion

### *ephrin-B1^+/−^* Mice as a Model for CFNS

In this study we have shown that, analogous to humans, heterozygous loss of *ephrin-B1* in mice results in defective development of the skull vault. Human CFNS patients carrying mutations in the *ephrin-B1* gene exhibit a range of craniofacial defects including ocular hypertelorism, malformation of the face (in particular the forehead and the nose), cranium bifidum occultum, and craniosynostosis [22]. Our study shows that at birth, *ephrin-B1^+/−^* mice exhibit a delay in the ossification of calvarial bones leading to a frontal foramen similar to cranium bifidum occultum, and in many instances, to a bifid nose, mimicking the human disease. Removing one copy of *ephrin-B2* resulted in increased severity of the frontal foramen, and an additional coronal suture defect in an *ephrin-B1^+/−^* background, but had no effect in *ephrin-B1* null embryos, suggesting that the lack of phenotype in *ephrin-B1* hemizygous males is not due to functional compensation by *ephrin-B2*. The effect of removing both copies of *ephrin-B2* on calvarial development could not be analyzed due to the early embryonic lethality of *ephrin-B2* null embryos.

Interestingly, our mutant mice did not exhibit craniosynostosis of the coronal sutures seen in humans. The processes leading to calvarial foramina and craniosynostosis are genetically linked, as evidenced by loss of function and gain of function mutations in the transcription factor Msx2, respectively. Moreover, heterozygosity for the transcription factor Twist can lead to craniosynostosis, as well as to foramina, and both transcription factors regulate differentiation of frontal bone osteoprogenitors (Ishii et al., 2003). Ectopic bone growth observed within the coronal suture of *ephrin-B1^+/−^/ephrin-B2^+/GFP^* embryos might be prescient of a premature fusion of the suture, but this could not be analyzed at later stages, since 100% of these animals die at birth (A. Davy, P. Soriano, unpublished data). The genetic interaction suggests, however, that the discrepancy in coronal suture phenotype between *ephrin-B1* heterozygous mice and humans could be due to genetic modifiers. Our expression analysis does not support a model in which wild-type expression of *ephrin-B1* controls suture formation by establishing boundaries in craniofacial mesenchyme. In fact, the observation that *ephrin-B1* null animals have no defects in calvarial bones argues that the function of *ephrin-B1* is not normally required for either calvarial bone development or proper suture formation. On the other hand, the fact that *ephrin-B1* is expressed in the meningeal layer could account for both the suture as well as the low-penetrance parietal bone phenotype observed in *ephrin-B1^+/−^* embryos because this layer is involved in suture and parietal bone formation [18,23].

Most of the mutations in *ephrin-B1* that have been identified in CFNS patients are located in the 5′ end of the gene and are consistent with loss-of-function mutations, either by introducing a premature stop codon, or by presumably interfering with the binding of ephrin-B1 to Eph receptors [9,10]. However, it was reported more recently that some patients harbored mutations in the 3′ end of the gene, which might affect specifically the reverse signaling activity of ephrin-B1 [24]. Our data using chimeric embryos demonstrate that a mutation in the PDZ binding domain of ephrin-B1, which is known to phenocopy some of the phenotypes observed in *ephrin-B1* null embryos including cleft palate [8], does not induce calvarial defects or polydactyly. These results indicate that the mutations found in CFNS patients might impinge on protein stability, protein localization, or binding of effector molecules independent of the PDZ domain.

### Defective Osteogenic Differentiation and Inhibition of GJC

Our results are consistent with a model in which inhibition of GJC at ectopic Eph/ephrin boundaries in *ephrin-B1^+/−^* females results in an abnormal differentiation of osteoprogenitors, thereby leading to the defective development of frontal bones and also, presumably, to polydactyly. Consistent with a previous report [6], we found that Eph/ephrin interaction inhibits GJC. In addition, junctional Cx43 was decreased at ectopic Eph/ephrin boundaries in vivo, and reduced junctional Cx43 correlated with decreased osteogenic differentiation of primary cells isolated from *ephrin-B1* heterozygous embryos. Finally, we found that overexpression of Cx43 partially rescued the calvarial phenotype observed in *ephrin-B1* heterozygote females. These results are consistent with other reports documenting the role of GJC and Cx43 in osteogenic differentiation [25–27]. In addition, several genetic studies have shown that defective regulation of GJC affects craniofacial and digit development, indicating that the structures affected in *ephrin-B1* heterozygous females are highly sensitive to alteration in GJC. Mice deficient for Cx43 exhibit delayed ossification of the calvarial bones and craniofacial abnormalities [28], but more importantly, mutations in *GJA1* (the gene coding for Cx43) in humans are responsible for oculodentodigital dysplasia (ODDD), a syndrome that is characterized by defective craniofacial development and digit formation [16]. An ENU screen in mice recently uncovered a dominant mouse mutation that exhibits many of the classic features of ODDD, and positional cloning revealed that these mice carry a mutation in *GJA1* that acts in a dominant-negative fashion to disrupt gap junction assembly and function [17]. On the basis of our results, we can not rule out the possibility that Eph/ephrin signaling might also impinge on osteogenic differentiation directly by activating signal transduction cascades (that would not involve the PDZ domain of ephrin-B1). However, the cytoplasmic kinases that are known targets of Eph/ephrin signaling (Src family kinases and MAPKs) have been shown to be positive regulators of osteogenic differentiation, which is not consistent with the inhibition of differentiation that we observe in vivo and in vitro. Moreover, we have not been able to detect a change in the level of MAPK activation in *ephrin-B1^+/−^* cultures (unpublished data).

We found that ephrin-B1 and Cx43 form a complex and that following engagement by Eph receptors, ephrin-B1 and Cx43 co-localize in clusters that could be indicative of endocytosis. Recent reports have shown that Eph/ephrins complexes are internalized following interaction [29–31], and endocytosis is also a well-known mode of regulation of connexons at the cell surface [32]. In both cases, pieces of the plasma membrane from neighboring cells are internalized. It is therefore conceivable that Cx43 might be co-internalized with Eph/ephrin complexes. However, it is also possible that endocytosis of connexons is regulated via a signal transduction cascade, since it has been shown recently that Eph/ephrin signaling regulates clathrin-mediated endocytosis by tyrosine phosphorylation of Synaptojanin 1 [33]. Tyrosine phosphorylation of Cx43 is also a mechanism by which GJC is regulated. In our pull-down assay, wild-type ephrin-B1 interacted preferentially with phosphorylated Cx43 whereas ephrin-B1^ΔPDZ^ interacted preferentially with unphosphorylated Cx43, suggesting that the interaction between ephrin-B1 and Cx43 might not be direct, and that these proteins might interact differently when at the cell surface or in the cytoplasm.

### Regulation of GJC and Cell Sorting

**Figure 9:**
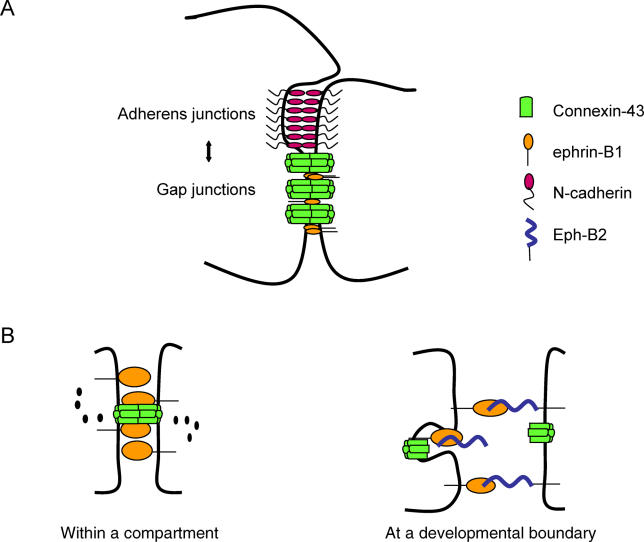
Mosaic Loss of ephrin-B1 and Establishment of Developmental Boundaries (A) Potential cross-talk between gap junctions and adherens junctions. Ephrin-B1 co-localizes with Cx43 in junctional plaques. Co-regulation of gap junctions and adherens junctions has been extensively studied. (B) Establishment of communication compartments for embryo patterning. Within a compartment and in the developing calvarial bones, all cells express ephrin-B1 and are coupled via GJC, exchanging second messengers (black dots). At a developmental boundary and at ectopic ephrin boundaries in *ephrin-B1^+/−^* embryos, Eph/ephrin interaction leads to inhibition of GJC, possibly through endocytosis of Cx43, concomitant with a loss of stable cell–cell interactions between the two cell types. Sorting between these cells and inhibition of GJC concur to establish distinct developmental compartments.

We observed that junctional Cx43 was enriched at interfaces between ephrin-B1–positive cells, suggesting that while GJC is inhibited at Eph/ephrin interfaces, it might be promoted at ephrin/ephrin interfaces. This observation, as well as the fact that overexpression of Cx43 leads to cell sorting, supports the idea that regulation of GJC contributes to Eph/ephrin-induced cell sorting. Although GJC has not been formally linked to cell sorting previously, there is ample evidence in the literature that regulation of GJC influences cell–cell contacts. Indeed, it has been shown in various experimental settings that inhibiting GJC, either through the use of blocking antibodies or dominant negative constructs, induces loss of adhesion [34–37]. In *Xenopus,* both overexpression of ephrin-B1 and dominant-negative connexin result in de-adhesion of the blastomeres [36,38]. Sorting of cells overexpressing Cx43 has also been reported previously in PC12 cells [39]. We therefore propose that regulation of GJC contributes to cell sorting downstream of Eph/ephrin interaction (Figure 9A).

A question that remains unanswered is whether or not the cell sorting observed in *ephrin-B1* heterozygotes is fully dependent on Eph receptors. Interestingly, an Eph-independent role for ephrin-B1 in regulating tight junctions has recently been reported [40], and it was recently shown that EphA4 can induce cell sorting independently of ephrins [41,42]. However, the fact that some CFNS patients harbor point mutations in the extracellular domain of ephrin-B1 that presumably have an effect on Eph/ephrin interaction argues for the involvement of Eph receptors in the sorting process.

On the basis of our data, we propose a model that explains the dominant effect of mosaic loss of ephrin-B1 in *ephrin-B1* heterozygous females, and that revisits the function of Eph/ephrin in embryo patterning (Figure 9B). In wild-type mice and within a developmental compartment, ephrin-expressing cells establish GJC which stabilizes cell–cell interactions and creates a communication compartment. In *ephrin-B1* heterozygotes as well as at a developmental boundary, GJC is inhibited between ephrin-positive and Eph-positive cells, possibly via endocytosis of Cx43, which prevents stable cell–cell interactions and leads to the formation of distinct compartments. Inhibition of GJC at developmental boundaries has been shown in a variety of models, including inter-rhombomeric boundaries [12,43], which also happen to be Eph/ephrin boundaries. In addition, overexpression of Cx43 has recently been shown to rescue a central nervous system boundary defect in mice [44].

In conclusion, our work demonstrates that in addition to their prominent role in regulating the actin cytoskeleton, Eph receptors and ephrins also play an important role in regulating gap junctional communication and suggests that improper regulation of GJC leads to the phenotypes observed in *ephrin-B1* heterozygous individuals. Together, these functions make Eph receptors and ephrins potent regulators of boundary formation and tissue patterning during embryonic development.

## Materials and Methods

### Mice.

*Ephrin-B1* mutant mice and CMV-Cx43 transgenic mice have been described elsewhere [8,22]. The *ephrin-B2^GFP^* allele was generated by inserting the H2BGFP cDNA cassette at the MluI site in the first exon of *ephrin-B2*. The MluI-XbaI fragment encompassing the start codon of the *ephrin-B2* gene was replaced with H2BGFP. The mutation was introduced in ES cells by homologous recombination. Mice were maintained in a 129S4/C57Bl6J mixed background. Genotyping was done by PCR using the following sets of primers: GFP-F: 5′-GCAAGAAGGCGGTGACTAAGGCGC-3′; GFP-R: 5′-GGCCGCCGCCAGTGCTTGAGGTCG-3′. Mice were housed in microisolator racks in a facility accredited by the Association for the Assessment and Accreditation of Laboratory Animal Care, and experimentation was reviewed by the Hutchinson Center Institutional Review Committee. The ES cells carrying the *ephrin-B1^ΔPDZ^* mutation have been described previously [8]. The sequence of the primer specific to the mutant allele was: 5′-GCCATGCTGGGCCTTCACT-3′. To obtain *ephrin-B1* null ES cells, one clone of targeted ES cells carrying the conditional allele of *ephrin-B1 (ephrin-B1^lox^)* was electroporated with a PGK-Cre expression vector. ES cell clones were isolated and screened by Southern-blot for the recombined *ephrin-B1* locus. Targeted ES cells were injected into blastocysts obtained from crosses between F1 (129S4/C57Bl6J) ROSA26-βgal homozygous males and either wild-type C57BL6J/CBAJ or MF1 females.

### X-gal staining and skeletal preparations.

Procedures used for X-gal staining and skeletal preparations have been described in detail elsewhere [8]. For the experiment with mice carrying the CMV-Cx43 transgene, quantification of the foramen area between frontal bones was performed using Photoshop (Adobe, San Jose, California, United States) to record the number of pixels corresponding to the foramen. The mean values for females with (97,901 pixels) and without (117,391 pixels) the transgene were then normalized to the mean value obtained for males (91,824 pixels) (no significant difference was found between males with or without the transgene, *n* = 10). Statistical significance was calculated using an unpaired *t*-test with Welch correction.

### In situ hybridization.

Section in situ hybridization experiments were performed on frontal sections of E14.5 embryos as described previously [45]. Whole-mount in situ hybridization was performed according to a protocol described elsewhere [46]. Probe sequences used for *ephrin-B1, EphB2,* and *EphB3* are available upon request.

### Primary cultures.

Presumptive frontal bone mesenchyme was dissected from E14.5 embryos and incubated with 1% trypsin/0.5% DNAse in PBS for 10 min at 37 °C. Trypsin was inactivated by addition of complete medium (DMEM containing 15% FCS) and cells were dissociated by trituration with a glass Pasteur pipette. Cells were pelleted by centrifugation and washed twice in complete medium. Cells were plated at high density in a 24-well plate and kept in culture in complete medium. After 3 d, cells were fixed in 2% PFA and rinsed three times in NTMT. AP activity was detected by incubating fixed cells with NBT/BCIP (Roche, Basel, Switzerland). Some cultures were further processed for immunofluorescence as described below. Quantification of AP activity was performed on digital images using the Image J software (http://rsb.info.nih.gov/ij). The data presented are representative of five independent experiments.

Primary NCCs were isolated from dissected branchial arches of E9.5 embryos as described above, except cells were cultured in F12 medium supplemented with 10% FCS.

### GJC assays.

NIH 3T3 cells were stably transfected with expression vectors for ephrin-B1, Eph-B2 receptor, or the pcDNA3 control vector. Recipient cells were plated at high density. Donor cells (NIH 3T3 expressing ephrin-B1 or primary NCCs) were incubated with calcein-AM (Molecular Probes, Eugene, Oregon, United States) for 20 min at 37 °C. Calcein-AM loaded cells (donors) were extensively washed in PBS, trypsinized, and dropped onto confluent monolayers of NIH 3T3 cells transfected with either pcDNA3 or expressing ephrin-B1 or various levels of Eph-B2. Transfer of calcein-AM through gap junctions was assessed after 3 h incubation at 37 °C. GJC establishment was quantified by two means for each conditions: (1) the percentage of cells that were spread and had transferred the dye versus cells that were spread but did not transfer the dye; and (2) the number of cells receiving the dye for each donor. These data were acquired by visual assessment either directly on the inverted microscope or from digital images. Statistical significance was calculated using a Student *t*-test.

### Western blot analysis.

Cells were scraped in 1% NP40 lysis buffer (50 mM Hepes [pH 7.5], 150 mM NaCl, 10% glycerol, 1.5 mM MgCl_2_, 1mM EGTA, 100 mM NaF), except for the experiment presented in Figure 7A (100 mM Tris [pH 7.4], 150 mM NaCl, 1mM EDTA). Protein lysates were incubated with either 5-μg EphB2-Fc or 4-μg Cx43 monoclonal antibody (generous gift from P. Lampe) for 4 h at 4 °C and subsequently with 20-μl ProteinA-Sepharose. Affinity complexes were analyzed by SDS-PAGE using the following antibodies: ephrin-B1 (A20 or C18, Santa Cruz Biotechnology, Santa Cruz, California, United States), Cx43 (Sigma, St. Louis, Missouri, United States). We have noted that the binding of Cx43 to ephrin-B1 is sensitive to the lysis buffer used: the interaction is lost in RIPA buffer.

### Immunofluorescence.

NIH 3T3 cells were plated on glass coverslips and transiently transfected with an expression vector for ephrin-B1. Forty hours after transfection, cells were either fixed in 2% PFA or incubated with 4-μg/ml EphB2-Fc for 30 min at 37 °C and then fixed in PFA. Cells were permeabilized with 0.1% Tx-100 for 3 min, rinsed in PBS, and incubated with a mix of EphB2-Fc (4 μg/ml) and either Cx43 monoclonal antibody or N-cadherin monoclonal antibody (Zymed, Carlsbad, California, United States). In primary cells, ephrin-B1 was detected using the 25H11 rat monoclonal antibody [47]. FITC- and Cy3-conjugated secondary antibodies were from Jackson ImmunoResearch (West Grove, Pennsylvania, United States). For the sorting experiments, HEK293T cells were transiently transfected with either DsRed or a Cx43 expression construct. Twenty-four hours after transfection, cells were trypsinized into a single-cell suspension and replated onto glass coverslips. Immunofluorescence was performed at 48 h post-transfection using a Cx43 antibody (Sigma).

For immunofluorescence on sections, paraffin sections were rehydrated and subjected to a citrate boil to reveal antigens. Tissue sections were blocked in Blocking solution (PBS/5% horse serum) 1 h at room temperature and incubated overnight at 4 °C in Cx43 antibody (1/100 in Blocking solution [Sigma]). Incubation with the Cy3-conjugated secondary antibody was for 1 h at room temperature (1/250 in Blocking solution). The number of Cx43 dots was counted on digital images acquired on a Delta Vision Deconvolution microscope (Applied Precision Inc., Issaquah, Washington, United States). The number of Cx43 dots was normalized to the number of junctions: for each section, we evaluated the number of wild-type/knock-out (KO) junctions (15–20) and counted a similar number of wild-type/wild-type and KO/KO junctions on each side of the wild-type/KO boundary.

## Supporting Information

Figure S1 Patterning of the Vibrissae Buds Is Altered in *ephrin-B1^+/−^* Embryos (A) Whole E14.5 embryos were stained with H&E to highlight whiskers buds of *ephrin-B1^Y/−^* hemizygous males (a) and *ephrin-B1^+/−^* heterozygous females (b). X-gal staining of E14.5 *ephrin-B1^+/lox^* embryo carrying the R26R and Wnt1-Cre alleles (c). The patterning of vibrissae buds is abnormal in heterozygote but not homozygote mutants. This defect is autonomous to the NCC lineage since eliminating *ephrin-B1* specifically in this lineage is sufficient to phenocopy the vibrissae bud defect (c).
(B) In situ hybridization on frontal sections of E14.5 wild-type embryo using probes for *ephrin-B1* (a) and *EphB2* (b) showing expression of these genes in the mesenchyme around vibrissae buds (arrowheads).
(3.0 MB PPT)

Figure S2 Calvarial Defects and Decreased Osteogenic Differentiation (A) Skeleton preparations of whole heads from E17.5 littermate embryos, *ephrin-B2^+/GFP^* single heterozygous mutants (a), *ephrin-B1^+/−^* single heterozygous females (b), and *ephrin-B1^+/−^/ephrin-B2^+/GFP^* double heterozygous females (c). Ectopic bone can be seen in the suture mesenchyme of *ephrin-B1/ephrin-B2* double heterozygous females (arrowhead in c) and the foramen between the frontal bones is larger than in *ephrin-B1^+/−^* embryos. An outline of the bones is presented for better visualization (d–f).
(B) AP staining on cryosections of E15.5 embryos. A wild-type (a), an *ephrin-B1* null male (b), and an *ephrin-B1^+/−^/ephrin-B2^+/GFP^* double heterozygous female (c) are shown. Bone front in the control embryo (a) and (b) is closer to the midline than bone front in the heterozygous littermate (c). In addition, the thickness of the bone is decreased in the *ephrin-B1^+/−^/ephrin-B2^+/GFP^* double heterozygous female. Both observations show that differentiation of the frontal bone is decreased in the heterozygote.
(2.2 MB PPT)

Figure S3 Ephrin-B1 and Cx43 Distribution in Primary Cultures and Differentiating Frontal Bones of *ephrin-B1^+/−^* Embryos (A) Ephrin-B1 was detected by immunofluorescence in cultures of primary mesenchymal cells (a) and (c) that were also stained for AP activity (b) and (d). Expression of ephrin-B1 was readily detected as a punctate staining on these primary cells, both in AP-positive (a) and (b) and in AP-negative cells (c) and (d).
(B) Western blot analysis of Cx43 levels in primary cultures of mesenchymal cells isolated from littermates of various genotypes. No difference in the overall Cx43 levels was detected.
(C) Paraffin sections from E12.5 control embryos *(ephrin-B1^Y/−^)* (a) and (b) and *ephrin-B1^+/−^* embryos (c) and (d) were stained for AP activity (a) and (c) and subsequently processed for Cx43 immunostaining (b) and (d). AP staining in the developing frontal bone of *ephrin-B1^+/−^* embryo is more irregular than in the control embryo, consistent with the results presented in Figure 3. However, no significant difference can be seen in the overall staining for Cx43 in this tissue.
(878 KB PPT)

Figure S4 *ephrin-B1^ΔPD^* ^Z^ Chimeric Embryos Do Not Exhibit Calvarial Defects (A) Skeletal preparations of whole heads from E18.5 *ephrin-B1^ΔPDZ^* chimeric embryos showing low (a) and high (b) contribution of mutant ES cells, assessed by PCR on tail DNA (c).
(B) Skeletal preparations of E18.5 wild-type (a), *ephrin-B1^−/−^* (b), and *ephrin-B1^ΔPDZ^* chimeras (c) and (d) show that chimeric embryos exhibit sternum defects reminiscent of *ephrin-B1* null embryos.
(1.1 MB PPT)

## Abbreviations

AP: alkaline phosphatase
CFNS: craniofrontonasal syndrome
Cx43: connexin43
ES: embryonic stem
GFP: green fluorescent protein
GJC: gap junction communication
KO: knock-out
NCC: neural crest cell
